# Supplementary figures and images for: Tissue-guided LASSO for prediction of clinical drug response using preclinical samples
Source: PLoS Comput Biol. 2020 Jan 22;16(1):e1007607. doi: 10.1371/journal.pcbi.1007607 (PMC6975549; doi:10.1371/journal.pcbi.1007607)

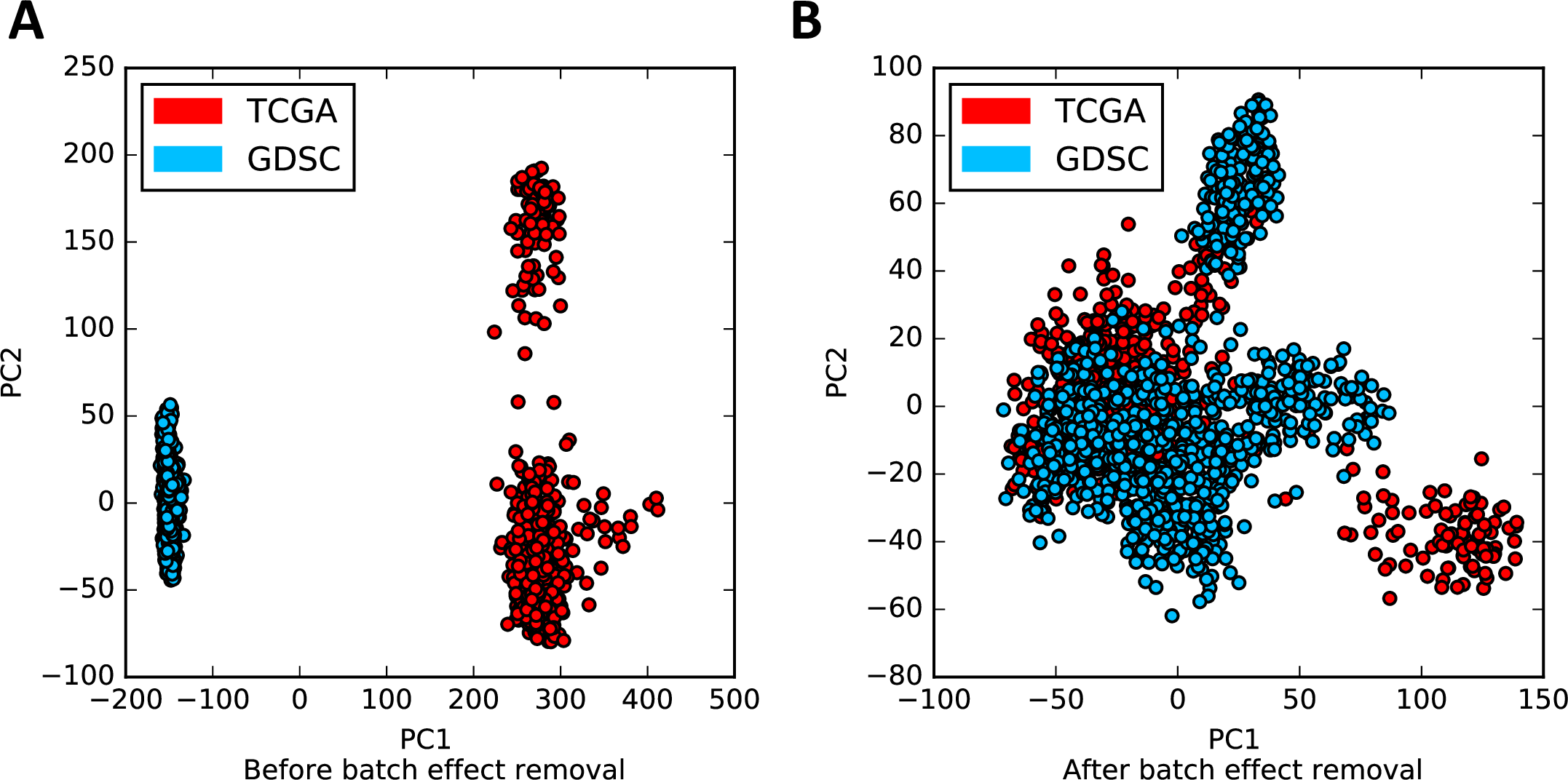

Supplement: S1 Fig — (TIF) [file pcbi.1007607.s013.tif]

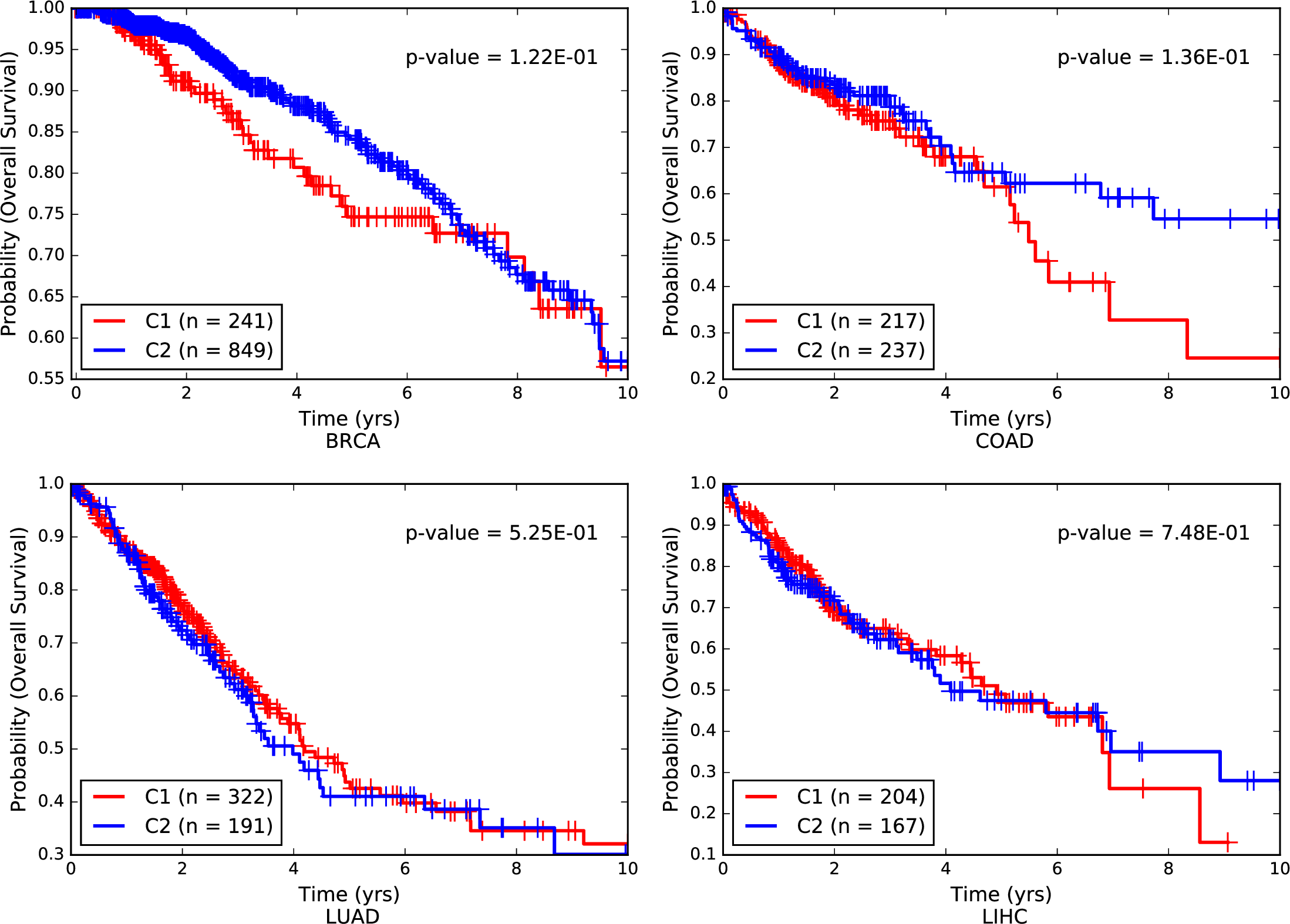

Supplement: S2 Fig — Patients were clustered into two groups using the expression of genes identified by TG-LASSO for more than 5 drugs in each tissue type. (TIF) [file pcbi.1007607.s014.tif]
